# Relationship between sleep characteristics and measures of body size and composition in a nationally-representative sample

**DOI:** 10.1186/s40608-016-0128-y

**Published:** 2016-11-11

**Authors:** Qian Xiao, Fangyi Gu, Neil Caporaso, Charles E. Matthews

**Affiliations:** 1 Department of Health and Human Physiology, University of Iowa, E118 Field House, Iowa City, Iowa 52242 USA; 2 Genetic Epidemiology Branch, Division of Cancer Epidemiology and Genetics, National Cancer Institute, National Institutes of Health, Rockville, Maryland USA; 3 Metabolic Epidemiology Branch, Division of Cancer Epidemiology and Genetics, National Cancer Institute, National Institutes of Health, Rockville, Maryland USA

**Keywords:** Sleep duration, Sleep quality, Snoring, Body composition

## Abstract

**Background:**

Short sleep has been linked to obesity. However, sleep is a multidimensional behavior that cannot be characterized solely by sleep duration. There is limited study that comprehensively examined different sleep characteristics in relation to obesity.

**Methods:**

We examined various aspects of sleep in relation to adiposity in 2005–2006 NHANES participants who were 18 or older and free of cardiovascular disease, cancer, emphysema, chronic bronchitis and depression (*N* = 3995). Sleep characteristics were self-reported, and included duration, overall quality, onset latency, fragmentation, daytime sleepiness, snoring, and sleep disorders. Body measurements included weight, height, waist circumference, and dual-energy X-ray absorptiometry measured fat mass.

**Results:**

Snoring was associated with higher BMI (adjusted difference in kg/m^2^ comparing snoring for 5+ nights/week with no snoring (95 % confidence interval), 1.85 (0.88, 2.83)), larger waist circumference (cm, 4.52 (2.29, 6.75)), higher percentage of body fat (%, 1.61 (0.84, 2.38)), and higher android/gynoid ratio (0.03 (0.01, 0.06)). The associations were independent of sleep duration and sleep quality, and cannot be explained by the existence of sleep disorders such as sleep apnea. Poor sleep quality (two or more problematic sleep conditions) and short sleep duration (<6 h) were also associated with higher measures of body size and fat composition, although the effects were attenuated after snoring was adjusted.

**Conclusion:**

In a nationally representative sample of healthy US adults, snoring, short sleep, and poor sleep quality were associated with higher adiposity.

**Electronic supplementary material:**

The online version of this article (doi:10.1186/s40608-016-0128-y) contains supplementary material, which is available to authorized users.

## Background

Growing evidence has linked sleep deficit with higher adiposity, and short sleep duration has been identified as an important risk factor for childhood and adult obesity [1]. Sleep is a complex, multidimensional behavior that cannot be characterized solely by sleep duration. Both insufficient quantity of sleep and poor sleep quality may lead to sleep deficiency. The 2011 NIH National Sleep Disorders Research Plan stated that “Sleep deficiency may result from prolonged wakefulness leading to sleep deprivation, insufficient sleep duration, sleep fragmentation, or a sleep disorder, such as in obstructive sleep apnea, that disrupts sleep and thereby renders sleep non-restorative” [2].

Several studies examined the relationship between obesity and different measures of sleep disturbances, sleepiness, and overall sleep quality, and they generally reported higher adiposity among people with poor sleep quality [3–11]. Moreover, multiple studies have also found a strong association between snoring and obesity [12–16]. However these studies have several limitations. First, none of the studies examined whether or not effects of sleep quantity, sleep quality and snoring are independent of each other. Additionally, all of these studies either only focused on one or a few aspects of sleep, or relied on a composite measure of sleep quality such as the Pittsburgh Sleep Quality Index (PSQI) without evaluating the effect of each individual component. Therefore, they were not able to provide information on which sleep characteristics had a stronger link with adiposity. Finally, almost all studies examined sleep characteristics in relation to body-mass index (BMI) alone, and none directly measured total and regional fat distribution.

We studied a comprehensive list of sleep characteristics in relation to anthropometric measures of adiposity as well as dual-energy X-ray absorptiometry (DXA) based measures of body composition in a nationally representative sample of the U.S. population. We put special emphasis on snoring, sleep duration as well as individual and overall measures of sleep quality. Our study aims to obtain a more detailed understanding of the relationship between sleep and adiposity.

## Methods

### Data source and study population

The NHANES is a cross-sectional survey designed to evaluate health and nutritional status of a representative sample of civilian noninstitutionalized US population, using a complex stratified multistage sampling design [17]. The survey is conducted by the National Center for Health Statistics of the US Centers for Disease Control and Prevention (Atlanta, Georgia). Our study utilized data from year 2005–2006, when both a questionnaire on sleep disorders and a DXA examination were included in the survey. Of the 6139 participants who completed the sleep disorder questionnaire, we focused our analysis on those who were 18 years or older (*N* = 5563). We further excluded those who had cancer (*N* = 420), cardiovascular disease (*N* = 450), emphysema (*N* = 35), or chronic bronchitis (*N* = 245), or those women who were pregnant (*N* = 349). Finally, we used the depression screener questionnaire to identify people with depression. We used a cut-off of ≥10 for moderate to severe depressive symptoms [18] and excluded participants with depression (*N* = 187). The final analytic sample included 3995 participants. We obtained all data and detailed survey protocols from the website of the National Center for Health Statistics [17]. The study was approved by the Centers for Disease Control and Prevention’s institutional review board.

### Assessment of sleep characteristics

We used the first 16 questions on the sleep questionnaire [19]. These sleep questions fall into five general categories: sleep duration, sleep quality, breathing problem during sleep, leg problems during sleep and history of sleep disorders. A full list of questions, values and categories, and labels used in this paper is presented in Additional file 1: Table S1. The population distribution of all sleep variables in the analytic sample is presented in Additional file 1: Table S2.

We evaluated sleep quality using a modified method by Bansil et al. [20]. We considered the following as having a condition suggesting poor sleep quality: reporting taking 1 h or more to fall asleep or “almost always” to any of the following six items: (1) having trouble falling asleep; (2) waking up during the night and having trouble getting back to sleep; (3) waking up too early in the morning and being unable to get back to sleep; (4) feeling unrested during the day, no matter how many hours of sleep were obtained; (5) feeling excessively or overly sleepy during the day; and (6) not getting enough sleep. We created a sleep quality index by counting the number of conditions each participant had that suggested poor sleep quality. Participants were divided into three categories based on their sleep quality index (0, 1 and 2 or more conditions suggesting poor sleep quality).

### Assessment of body size and composition

Standing height, weight and waist circumference were measured and whole body DXA scans were performed using a Hologic QDR 4500 fan beam densitometer (Hologic Inc., Bedford MA). DXA scans were administered to participants who were 8–69 years of age, excluding those who were pregnant, reported radiographic contrast material (barium) exam in the past 7 days, or reported a weight over 300 lb or a height of 6 ft 5 in. or more. The scan for each participant was analyzed by the University of California-San Francisco, department of radiology, using standard radiologic techniques and study specific protocols developed for NHANES.

We examined four anthropometric and DXA measurements in our study, including two measurements of overall adiposity, BMI (calculated by dividing weight by height squared (kg/m^2^)) and percent body fat (calculated as total fat mass divided by body weight), as well as two measurements of central adiposity, waist circumference and android/gynoid fat ratio. The android area is roughly the area around the waist between the mid-point of the lumbar spine and the top of the pelvis; the gynoid area lies roughly between the head of the femur and mid-thigh. A high value of android to gynoid fat ratio suggests high truncal adiposity, and has been linked with risk of diseases [21]. Because the missingness of DXA data was not completely at random and was associated with age, weight, and height, multiple imputation was performed to reduce potential bias. Detailed protocols describing the methods have been published [19, 22].

### Statistical analysis

Because the distributions of body measurements are presumed normal, we used Pearson correlation coefficients to evaluate their correlations. For sleep variables, which were categorical, we used the nonparametric Spearman’s rank correlation coefficients to evaluate their correlations. Because our outcome variables (body measurements) were continuous variables, we used multiple linear regression models to estimate the difference in measurements of body size and composition comparing non-reference categories of sleep variables to the reference categories (reference categories for each sleep variable are presented in Additional file 1: Table S1). We ran regression analysis for men and women separately as well as with both sexes combined. Because no statistically significant interaction was detected with sex, we present results from combined analysis for our main tables. The multivariate models were adjusted for age (continuous), sex (male, female), race/ethnicity (Mexican American, other Hispanic, non-Hispanic white, non-Hispanic black, other), education (less than 9^th^ grade, 9–11^th^ grade, high school/GED, some college or AA degree, college and higher), smoking status (Never, former, current smoker with <1 pck/d, current smoker with 1+ pck/d, missing), alcohol consumption (non-drinker, <1, 1- < 3, 3- < 7 drink/week, 1- < 2, 2+ drink/day, missing), intakes of fat (continuous), carbohydrate (continuous) and total calories (continuous), daily physical activity pattern (“do not walk about very much”, “stand or walk about a lot but do not carry or lift things”, “lift light load or climb stairs or hills often”, “do heavy work or carry heavy loads”) and diabetes (yes, no). Missing values for each variable were coded as a separate category. Information on smoking status and alcohol drinking were only available for people who were age 20 or older. In a sensitivity analysis, removing participants who were 18 or 19 years old did not change the results substantially. Analysis of total body fat utilized five imputed datasets [22]. Although the main results were presented for each category of sleep variables, we also modeled sleep variables as continuous and evaluated the coefficients using a Wald test to test for trend of associations. To account for the complex survey design, survey nonresponse, and post-stratification in NHANES, we employed appropriate survey sampling weights in all statistical analyses. All analyses were performed using SAS (SAS Institute, Cary, North Carolina).

## Results

The correlations among the sleep variables were generally low to moderate (absolute value <0.6, Additional file 1: Table S3). Most of the measures of body size and composition were moderately to highly correlated, except for percent body fat and android/gynoid fat ratio (Additional file 1: Table S4). Study characteristics by sleep duration, snoring and sleep quality are presented in Table 1 (by sleep duration) and Additional file 1: Table S5 (by snoring) and Additional file 1: Table S6 (by sleep quality).

**Table 1:** Study participant characteristics by sleep duration, NHANES 2005–2006

	Sleep duration				
	≤56 h	6 h	7 h	8 h	≥9 h
Age, year, mean (SD)	42.1 (15.9)	42.9 (15.0)	42.4 (15.1)	43.2 (16.9)	41.7 (20.7)
Female, %	39.4	47.3	49.7	49.3	57.3
Non-Hispanic White, %	58.6	67.0	73.6	71.9	68.8
Non-Hispanic Black, %	23.5	14.9	8.4	8.9	12.3
Less than high school grad, %	20.2	13.4	14.5	15.0	26.2
Current smoker, %	34.8	25.4	17.8	18.6	27.1
Former smoker, %	16.7	20.0	23.4	24.1	16.9
Alcohol consumption, > 1 drink/day, %	15.1	14.1	12.5	14.8	11.3
Fat intake, mean (SD), gram/kcal	37.2 (8.4)	37.7 (8.8)	37.3 (8.5)	37.1 (8.2)	37.1 (7.9)
Carbohydrate intake, mean (SD), gram/kcal	124 (23)	121 (25)	121 (23)	121 (23)	124 (24)
Total caloric intake, kcal	2256 (934)	2186 (864)	2224 (852)	2176 (866)	2084 (870)
History of diabetes, %	8.1	5.2	4.3	6.3	7.1
Sit during the day and not walk about much, %	21.0	18.7	21.1	20.7	26.9

Percentages, means and standard deviations are weighted

Snoring was positively associated with all body measurement (Table 2), and the relationship remained statistically significant after adjusting for multiple factors (*p-for-trend*: *<.0001*). Even those participants who reported snoring only 1–2 nights/week had significantly higher overall and central adiposity. Because the relationship between snoring and adiposity may be impacted by sleep-disordered breathing, such as obstructive sleep apnea, we performed sensitivity analysis restricting to those reporting no sleep disorder and rare/never snorting (≤2 nights/week). After excluding people with sleep disorder and who report snort frequently (~15 % of the study population), the effect estimates for the highest category of snoring (5+ nights/week) was attenuated by 15–20 % but remained significant, while changes in the results for other snoring categories and p-for-trend were minimal (data not shown).

**Table 2:** Associations between snoring and measurements of body size and composition, NHANES 2005–2006

	Snoring frequency, nights/week				*p for trend*
β (95 % CI)	5+	3–4	1–2	Never	
BMI (kg/m^2^)					
age and sex adjusted	4.59 (3.52, 5.67)	3.02 (2.29, 3.75)	1.85 (0.85, 2.86)	ref	*<.0001*
multivariable^a^	4.56 (3.56, 5.55)	2.80 (2.01, 3.60)	1.85 (0.88, 2.83)	ref	*<.0001*
multivariable^b^	4.45 (3.44, 5.46)	2.70 (1.89, 3.51)	1.90 (0.95, 2.84)	ref	*<.0001*
Percent body fat					
age and sex adjusted	3.47 (2.47, 4.48)	2.51 (1.82, 3.21)	1.49 (0.64, 2.34)	ref	*<.0001*
multivariable^a^	3.57 (2.69, 4.45)	2.46 (1.83, 3.10)	1.61 (0.84, 2.38)	ref	<.0001
multivariable^b^	3.48 (2.59, 4.36)	2.38 (1.73, 3.03)	1.65 (0.88, 2.41)	ref	<.0001
Waist circumference (cm)					
age and sex adjusted	10.56 (8.20, 12.91)	7.71 (5.77, 9.65)	4.65 (2.29, 7.00)	ref	*<.0001*
multivariable^a^	10.29 (8.09, 12.48)	7.18 (5.14, 9.23)	4.52 (2.29, 6.75)	ref	*<.0001*
multivariable^b^	10.04 (7.84, 12.25)	6.92 (4.86, 8.99)	4.59 (2.40, 6.77)	ref	*<.0001*
Android/Gynoid fat ratio					
age and sex adjusted	0.10 (0.07, 0.14)	0.09 (0.07, 0.12)	0.03 (0.01, 0.05)	ref	*<.0001*
multivariable^a^	0.10 (0.07, 0.13)	0.09 (0.07, 0.12)	0.03 (0.01, 0.06)	ref	*<.0001*
multivariable^b^	0.10 (0.07, 0.13)	0.09 (0.07, 0.12)	0.03 (0.01, 0.06)	ref	*<.0001*

^a^adjusted for age, sex, race/ethnicity, smoking status, alcohol drinking, education, intakes of fat, carbohydrate and total calories, physical activity and diabetes

^b^adjusted for covariates in ^a^as well as sleep duration and sleep quality index

Reporting more sleep conditions that suggested poor sleep quality was associated with higher overall adiposity and central adiposity (Table 3). Compared to those who reported no indicator of a sleep condition, participants who reported two or more conditions had higher BMI (kg/m^2^, multivariate-adjusted mean difference (95 % CI), 1.19 (0.34, 2.04)), higher percentage of body fat (1.18 (0.44, 1.93), larger waist circumference (cm, 2.82 (0.87, 4.77)), and higher android/gynoid fat ratio (0.04 (0.00, 0.08). The associations with individual sleep quality variables were less consistent and generally null (Additional file 1: Table S7).

**Table 3:** Associations between sleep quality and measurements of body size and composition, NHANES 2005–2006

	Sleep quality score			*p for trend*
β (95 % CI)	2+	1	0	
BMI (kg/m^2^)				
age and sex adjusted	1.29 (0.40, 2.18)	1.10 (0.34, 1.85)	ref	*0.002*
multivariable^a^	1.19 (0.34, 2.04)	0.93 (0.30, 1.57)	ref	*0.002*
multivariable^b^	0.55 (−0.41, 1.51)	0.87 (0.24, 1.49)	ref	*0.05*
Percent body fat				
age and sex adjusted	1.06 (0.29, 1.84)	0.84 (0.20, 1.48)	ref	*0.002*
multivariable^a^	1.18 (0.44, 1.93)	0.87 (0.22, 1.53)	ref	*0.0004*
multivariable^b^	0.82 (0.11, 1.53)	0.78 (−0.07, 1.63)	ref	*0.01*
Waist circumference (cm)				
age and sex adjusted	3.37 (1.40, 5.33)	1.91 (0.19, 3.62)	ref	*0.0003*
multivariable^a^	2.82 (0.87, 4.77)	1.59 (0.06, 3.12)	ref	*0.001*
multivariable^b^	1.77 (−0.65, 4.19)	1.30 (−0.38, 2.99)	ref	*0.01*
Android/Gynoid fat ratio				
age and sex adjusted	0.03 (0.00, 0.07)	0.01 (−0.01, 0.04)	ref	*0.02*
multivariable^a^	0.04 (0.00, 0.08)	0.02 (−0.01, 0.04)	ref	*0.01*
multivariable^b^	0.03 (0.00, 0.06)	0.01 (−0.03, 0.04)	ref	*0.03*

^a^adjusted for age, sex, race/ethnicity, smoking status, alcohol drinking, education, intakes of fat, carbohydrate and total calories, physical activity and diabetes

^b^adjusted for covariates in ^a^as well as snoring and sleep duration

We found that short sleep duration (≤5 h) was associated with higher values of all body measurements, except for android/gynoid fat ratio (Table 4). We also observed a dose effect of sleep duration on these measurements. Compared with the 8 h group, the increase in body measurements for the ≤5 h group was almost twice as high as that for the 6 h group, while the increase for the 7 h group was slight and not statistically significant. Long sleep (≥9 h) was not associated with body size or adiposity.

**Table 4:** Associations between sleep duration and measurements of body size and composition, NHANES 2005–2006

	Sleep duration, hour					*p for trend*
β (95 % CI)	≤5	6	7	8	≥9	
BMI (kg/m^2^)						
age and sex adjusted	2.20 (0.87, 3.52)	1.19 (0.37, 2.01)	0.41 (−0.31, 1.13)	ref	0.29 (−1.37, 1.95)	*0.001*
multivariable^a^	1.82 (0.66, 2.98)	1.07 (0.36, 1.78)	0.45 (−0.23, 1.13)	ref	0.20 (−1.32, 1.73)	*0.001*
multivariable ^b^	1.55 (0.28, 2.82)	0.64 (−0.10, 1.38)	0.24 (−0.47, 0.95)	ref	0.23 (−1.26, 1.72)	*0.005*
Percent body fat						
age and sex adjusted	1.35 (0.23, 2.48)	0.46 (−0.42, 1.34)	0.34 (−0.28, 0.96)	ref	1.02 (−0.52, 2.57)	*0.18*
multivariable^a^	1.48 (0.51, 2.45)	0.54 (−0.15, 1.24)	0.47 (−0.07, 1.00)	ref	1.06 (−0.39, 2.52)	*0.07*
multivariable^b^	1.09 (0.15, 2.04)	0.09 (−0.64, 0.82)	0.22 (−0.40, 0.84)	ref	0.79 (−0.07, 1.63)	*0.36*
Waist circumference (cm)						
age and sex adjusted	4.28 (1.18, 7.38)	1.84 (−0.11, 3.79)	0.78 (−0.82, 2.38)	ref	0.02 (−3.21, 3.26)	*0.006*
multivariable^a^	3.69 (0.91, 6.46)	1.71 (0.10, 3.31)	0.95 (−0.54, 2.44)	ref	−0.23 (−3.25, 2.79)	*0.004*
multivariable^b^	3.15 (−0.07, 4.19)	1.30 (−0.38, 2.99)	0.49 (−0.91, 1.88)	ref	−0.34 (−2.95, 2.26)	*0.04*
Android/Gynoid fat ratio						
age and sex adjusted	0.02 (−0.02, 0.05)	0.00 (−0.04, 0.03)	−0.01 (−0.03, 0.01)	ref	−0.02 (−0.05, 0.02)	*0.35*
multivariable^a^	0.02 (−0.02, 0.06)	0.00 (−0.03, 0.03)	−0.01 (−0.03, 0.02)	ref	−0.02 (−0.06, 0.01)	*0.08*
multivariable^b^	0.00 (−0.03, 0.04)	−0.01 (−0.04, 0.01)	−0.02 (−0.04, 0.01)	ref	−0.02 (−0.06, 0.02)	*0.74*

^a^adjusted for age, sex, race/ethnicity, smoking status, alcohol drinking, education, intakes of fat, carbohydrate and total calories, physical activity and diabetes

^b^adjusted for covariates in ^a^as well as snoring and sleep quality

Additionally, we assessed the associations of snoring, poor sleep quality, and sleep duration with body size and composition in models mutually adjusted for these three sleep variables (Tables 2, 3 and 4, multivariate model b). We found that adjusting for sleep quality and sleep duration had a minimal impact on the association between snoring and higher adiposity. In contrast, the associations with sleep duration and sleep quality index were substantially attenuated in mutually adjusted models, although the trend and direction of the associations remain similar.

Finally, we examined sleep disorders in relation to adiposity. Reporting any sleep disorder was associated with greater body size and higher DXA measurements of body composition (Additional file1: Table S7), and it was largely driven by sleep apnea. Participants with restless leg syndrome also had higher values for body measurements, but due to small numbers, the associations were not statistically significant. Insomnia was not associated with any of the body measurements.

## Discussion

In a nationally-representative population, we found that snoring, short sleep duration, and poor sleep quality are all associated with higher adiposity. Among the three, snoring had the strongest association with adiposity. The association is independent of sleep duration and sleep quality, and cannot be explained by the existence of sleep disorders such as sleep apnea. In contrast, the relationship between higher adiposity and poor sleep quality and short sleep appeared to be partially explained by snoring and the other sleep characteristics.

Our finding of a positive relationship between snoring frequency and adiposity is consistent with previous literature that repeatedly linked snoring with higher BMI and larger waist circumference [12–16]. Snoring is an important indicator of sleep-disordered breathing (SDB), and there is a well-established association between SDB and obesity [23]. The relationship between SDB and obesity is bidirectional [24]. Increasing body fat, particularly fat accumulation around the neck and in the abdomen, can make the upper airway vulnerable to collapse during sleep and lead to the development of SDB, such as obstructive sleep apnea. On the other hand, recent evidence has suggested that SDB can lead to elevated oxidative stress and chronic inflammation due to hypoxia, which may subsequently contribute to the development of obesity and metabolic dysfunction [25]. In our study, even occasional snoring (1–2 days/week) was associated with higher adiposity, and the relationship remains after removing participants reporting sleep disorder and frequent snort. There were few studies that distinguished simple snoring from more severe forms of SDB, such as obstructive sleep apnea, and examined the relationship between simple snoring and obesity. However, several previous studies have also found that occasional snoring (1–3 day/week) was associated with central obesity and metabolic syndrome in Korean population [14] and women who reported “snoring occasionally” had a higher risk of developing diabetes in the Nurses’ Health Study [26]. These findings, together with ours, suggest that the prevalence of obesity and obesity–related conditions may be higher even among occasional snorers.

There is a well-documented association between short sleep and higher BMI [1], and we confirmed this association in our study. Particularly, we found that the increase in body size and adiposity associated with short sleep was much higher beyond the <6 h threshold, suggesting a strong link between sleep deficiency and excessively high adiposity. Several mechanisms have been proposed to account for the association [27]. Insufficient sleep has been associated with endocrine alterations that result in enhanced hunger and appetite (e.g. increased levels of ghrelin and cortisol, decreased levels of leptin and peptide YY, and impaired insulin sensitivity) [28–31]. Moreover, earlier studies have also reported increase in caloric intake among short sleepers [32] and following sleep restriction [33]. In addition to promoting eating, some of the hormonal changes associated with short sleep may also stimulate energy storage and fat accumulation [34], which may also lead to development of obesity. The association between long sleep duration and obesity is controversial. Some studies suggested a U-shaped association between sleep duration and obesity [35]. In our study, participants who reported more than 8 h of sleep did not have significantly higher measures of adiposity; however, we are limited in statistical power to examine more extreme long sleep duration in relation to adiposity.

We observed that poor sleep quality was associated with higher adiposity. This is consistent with previous studies, all of which reported an association between higher BMI and poor sleep quality [3–11]. Like short sleep duration, poor sleep quality has also been linked with increased hunger or greater desire to eat and changes in appetite-regulating hormones [36, 37], suggesting that sleep deficiency caused by either insufficient sleep or poor sleep quality may activate similar biological mechanisms that regulate energy intake and result in higher adiposity. Interestingly, we found that compared to the composite sleep index, individual variables on sleep quality had much weaker and less consistent association with adiposity. Several factors may explain this discrepancy. First, we are limited by sample size and may not be able to detect statistically significant associations for each category of the individual sleep variables. Second, the effect associated with individual sleep variables may be diluted by other sleep conditions (for example, the reference group for one sleep condition may include people who had other sleep conditions that may affect adiposity). Finally, we observed a dose effect that people who reported 2 or more conditions that suggested poor sleep quality had the highest adiposity. This may suggest that disturbances in individual sleep aspects may have a stronger association with adiposity than overall sleep quality has, or information on single sleep variables may not accurately reflect overall sleep quality.

A unique contribution of our study is that we examined whether the associations of adiposity with snoring, sleep quality and sleep duration were independent of each other in mutually adjusted models. No previous study attempted to examine all three aspects of sleep simultaneously. Among the few studies that collected information on different sleep characteristics, only a minority of them attempted to control for other sleep aspects. For example, three studies adjusted for sleep duration [5, 9, 11] and one study adjusted for snoring [6] in their analysis on sleep quality and adiposity, and reported that the association between poor sleep quality and higher BMI remained after the additional adjustment. In our study, after simultaneously including all three sleep variables in the model, we found that snoring remained strongly associated with adiposity regardless of sleep duration and quality, suggesting that this relationship is robust and may involve distinct mechanisms. In contrast, we found the associations with sleep duration and sleep quality were attenuated in mutually adjusted models, and this may be explained by the possibility that insufficient sleep and sleep disturbances may share some common pathways, with each other and with snoring, that mediate their associations with adiposity.

Another strength of our study is examining sleep in relation to both anthropometric measurements and DXA measured body composition. We found that overall the associations with sleep characteristics were similar among different measurements of body adiposity. This is consistent with previous studies that reported that anthropometric measures, such as BMI and waist circumference, are generally good indicators of adiposity and correlate well with DXA measured body composition [38]. However, we found that short sleep was not associated with android/gynoid fat ratio, although it was associated with all other body measurements, including waist circumference, the other measure of central adiposity. Although no other studies examined sleep in relation to android/gynoid fat ratio, earlier reports showed an inverse association between sleep duration and waist-to-hip ratio [30, 39], a substitute measure of android/gynoid fat ratio. It is unclear what caused this discrepancy and more studies are needed to clarify this relationship.

There are several limitations of our study. First, this is a cross-sectional study with both sleep information and body measurements obtained at the same time. Therefore we are not able to determine the temporal relationship between them or make causal inference. Second, all sleep variables were self-reported and are therefore subject to reporting errors and possible bias. Also, self-reported sleep characteristics cannot accurately capture important parameters of sleep architecture, such as the duration of rapid eye movement sleep and slow wave sleep, which has been linked to BMI and waist circumference independent of total sleep duration [6]. Objective measurement of sleep, such as atigraphy and polysomnography, may be used to better assess these sleep characteristics. Moreover, we do not have information on circadian patterns, such as chronotype, the regularity of circadian rhythm, and social jet lag which have been shown to be important in regulating energy balance and associated with obesity [5, 40–42]. Additionally, participants who had a self-reported weight of >300 lb and/or height of >6′5" were excluded from DXA scans and we weren’t able to examine body composition in this population, which prevented us from studying the relationship between sleep variables and more extreme body types. Lastly, although we adjusted for potential confounders, residual confounding remains a possibility, as in all observational studies.

## Conclusions

In summary, in this nationally represented population, we found snoring is strongly associated with adiposity. Moreover, both insufficient sleep duration and poor sleep quality are also associated with larger body size and higher adiposity. Our findings suggest that the prevalence of obesity might be disproportionately high among people with sleep deficiency.

## Additional file

Additional file 1: Table S1. Questions on sleep habits and sleep disorders in the sleep disorder questionnaire, NHANES 2005–2006. **Table S2.** Population distribution by sleep characteristic in the analytic sample, NHANES 2005–2006. **Table S3.** Spearman correlation coefficient ^a^ among sleep variables, NHANES 2005–2006. **Table S4.** Spearman correlation coefficient ^a^ among measurements of adiposity, by sex, NHANES 2005–2006. **Table S5.** Study participant characteristics by snoring, NHANES 2005–2006. **Table S6.** Study participant characteristics by sleep quality, NHANES 2005–2006. **Table S7.** Multivariate ^a^ associations between sleep characteristics and measurements of adiposity in men, NHANES 2005–2006. (DOCX 51 kb)

## Abbreviations

BMI: Body-mass index
CIs: Confidence intervals
SD: Standard deviation
