# Supplementary material for: Relationship between sleep characteristics and measures of body size and composition in a nationally-representative sample
Source: BMC Obes. 2016 Nov 11;3:48. doi: 10.1186/s40608-016-0128-y (PMC5106827; doi:10.1186/s40608-016-0128-y)
Supplement: Additional file 1: Table S1. — Questions on sleep habits and sleep disorders in the sleep disorder questionnaire, NHANES 2005–2006. Table S2. Population distribution by sleep characteristic in the analytic sample, NHANES 2005–2006. Table S3. Spearman correlation coefficient a among sleep variables, NHANES 2005–2006. Table S4. Spearman correlation coefficient a among measurements of adiposity, by sex, NHANES 2005–2006. Table S5. Study participant characteristics by snoring, NHANES 2005–2006. Table S6. Study participant characteristics by sleep quality, NHANES 2005–2006. Table S7. Multivariate a associations between sleep characteristics and measurements of adiposity in men, NHANES 2005–2006. (DOCX 51 kb) [file 40608_2016_128_MOESM1_ESM.docx]

Additional file 1

**Table S1** Questions on sleep habits and sleep disorders in the sleep disorder questionnaire, NHANES 2005-2006

| **Questions** | **Labels used in this study** | **Values** |
| --- | --- | --- |
| ***Sleep duration*** |  |  |
| How much sleep do you usually get at night no weekdays or workdays? | Sleep duration | Originally reported in hours and grouped into ≤5, 6, 7, 8 (ref), ≥9hr for analysis |
| ***Sleep quality*** |  |  |
| How long does it usually take you to fall asleep at bed time? | How long to fall asleep | Originally reported in minutes and grouped into ≥1 hr, 30 min-<1 hr, 15-<30 min and <15 min (ref) for analysis |
| In the past month, how often did you have trouble falling asleep | Have trouble falling asleep | Never (ref), rarely (1 times/month), sometimes (2-4 times/month), often (5-15 times/month), almost always (16-30 times/month) |
| In the past month, how often did you wake up during the night and had trouble getting back to sleep? | Wake up at night | Never (ref), rarely (1 times/month), sometimes (2-4 times/month), often (5-15 times/month), almost always (16-30 times/month) |
| In the past month, how often did you wake up too early in the morning and were unable to get back to sleep? | Wake up too early | Never (ref), rarely (1 times/month), sometimes (2-4 times/month), often (5-15 times/month), almost always (16-30 times/month) |
| In the past month, how often did you feel unrested during the day, no matter how many hours of sleep you had? | Unrest during the day | Never (ref), rarely (1 times/month), sometimes (2-4 times/month), often (5-15 times/month), almost always (16-30 times/month) |
| In the past month, how often did you feel excessively or overly sleepy during the day? | Overly sleepy | Never (ref), rarely (1 times/month), sometimes (2-4 times/month), often (5-15 times/month), almost always (16-30 times/month) |
| In the past month, how often did you not get enough sleep? | Not enough sleep | Never (ref), rarely (1 times/month), sometimes (2-4 times/month), often (5-15 times/month), almost always (16-30 times/month) |
| In the past month, how often did you take sleeping pills or other medication to help you sleep? | Take sleeping pills | Never (ref), rarely (1 times/month), sometimes (2-4 times/month), often (5-15 times/month), almost always (16-30 times/month) |
| ***Breathing problem during sleep*** |  |  |
| In the past 12 months, how often did you snore while you were sleeping? | Snoring | Never (ref), rarely (1-2 nights/week), occasionally (3-4 nights/week), frequently (5+ nights/week) |
| In the past 12 months, how often did you snort, gasp, or stop breathing while you were sleeping? | Snort | Never (ref), rarely (1-2 nights/week), occasionally (3-4 nights/week), frequently (5+ nights/week) |
| ***Leg problem during Sleep*** |  |  |
| In the past month, how often did you have leg jerks while trying to sleep? | Leg jerks | Never (ref), rarely (1 times/month), sometimes (2-4 times/month), often (5-15 times/month), almost always (16-30 times/month) |
| In the past month, how often did you have leg cramps while trying to sleep? | Leg cramps | Never (ref), rarely (1 times/month), sometimes (2-4 times/month), often (5-15 times/month), almost always (16-30 times/month) |
| ***Sleep disorder*** |  |  |
| Have you ever told a doctor or other health professional that you have trouble sleeping? | Have trouble sleeping | Yes, no (ref) |
| Have you ever been told by a doctor or other health professional that you have a sleep disorder? | Any sleep disorder | Yes, no (ref) |
| What was the sleep disorder? |  |  |
| Sleep Apnea | Apnea | Yes, no (ref) |
| Insomnia | Insomnia | Yes, no (ref) |
| Restless legs | Restless legs | Yes, no (ref) |
| Other | Other sleep disorder | Yes, no (ref) |

**Table S2** Population distribution by sleep characteristic in the analytic sample, NHANES 2005-2006

|  | **Participants with BMI,** | **Participants with waist circumference,**  **N (%)** ^a^ | **Participants with DXA,** |
| --- | --- | --- | --- |
|  | **N (%)** ^a^ |  | **N (%)** ^a^ |
| **Sleep Duration** |  |  |  |
| ≤5 hr | 497 (11.5) | 482 (11.6) | 368 (10.9) |
| 6 hr | 868 (23.5) | 840 (23.4) | 675 (23.7) |
| 7 hr | 1100 (32.9) | 1066 (33.0) | 865 (33.7) |
| 8 hr | 983 (26.0) | 956 (26.1) | 748 (26.1) |
| ≥9 hr | 277 (12.3) | 263 (6.0) | 192 (5.6) |
| **Sleep Quality** |  |  |  |
| **Sleep quality index** |  |  |  |
| 2+ | 436 (12.0) | 424 (12.2) | 322 (11.6) |
| 1 | 540 (13.2) | 513 (12.9) | 399 (12.6) |
| 0 | 2726 (74.8) | 2648 (74.9) | 2112 (75.8) |
| **How long to fall asleep** |  |  |  |
| >= 1hr | 496 (11.6) | 472 (11.5) | 359 (10.9) |
| 30-<1 hr | 809 (21.0) | 793 (21.2) | 633 (21.1) |
| 15-<30 min | 709 (19.5) | 684 (19.5) | 555 (19.8) |
| <15 min | 1703 (47.9) | 1651 (47.8) | 1297 (48.1) |
| **Trouble fall asleep** |  |  |  |
| Almost always (16-30/mo) | 201 (5.4) | 194 (5.5) | 146 (5.0) |
| Often (5-15/mo) | 270 (7.6) | 265 (7.7) | 206 (7.6) |
| Sometimes (2-4/mo) | 766 (22.1) | 742 (22.0) | 588 (21.9) |
| Rarely (1/mo) | 776 (23.2) | 758 (23.4) | 608 (23.7) |
| Never | 1714 (41.6) | 1650 (41.4) | 1301 (41.8) |
| **Wake up at night** |  |  |  |
| Almost always (16-30/mo) | 196 (5.4) | 192 (5.5) | 143 (5.1) |
| Often (5-15/mo) | 385 (12.1) | 376 (12.1) | 286 (11.9) |
| Sometimes (2-4/mo) | 819 (23.2) | 790 (23.2) | 615 (23.0) |
| Rarely (1/mo) | 770 (21.7) | 759 (22.0) | 625 (22.9) |
| Never | 1554 (37.6) | 1489 (37.3) | 1178 (37.1) |
| **Wake up too early in morning** |  |  |  |
| Almost always (16-30/mo) | 174 (4.7) | 167 (4.7) | 124 (4.4) |
| Often (5-15/mo) | 339 (10.2) | 329 (10.2) | 261 (10.5) |
| Sometimes (2-4/mo) | 705 (18.9) | 691 (19.0) | 543 (19.0) |
| Rarely (1/mo) | 730 (21.7) | 716 (21.8) | 577 (21.9) |
| Never | 1775 (44.6) | 1702 (44.3) | 1343 (44.2) |
| **Feeling unrest during the day** |  |  |  |
| Almost always (16-30/mo) | 262 (7.2) | 255 (7.2) | 193 (6.9) |
| Often (5-15/mo) | 504 (15.3) | 493 (15.4) | 406 (15.3) |
| Sometimes (2-4/mo) | 1086 (31.4) | 1055 (31.5) | 859 (32.2) |
| Rarely (1/mo) | 649 (19.0) | 632 (19.0) | 515 (19.9) |
| Never | 1223 (27.1) | 1171 (26.9) | 874 (25.7) |
| **Not enough sleep** |  |  |  |
| Almost always (16-30/mo) | 306 (8.3) | 295 (8.3) | 232 (8.0) |
| Often (5-15/mo) | 545 (15.7) | 531 (15.8) | 437 (16.3) |
| Sometimes (2-4/mo) | 1085 (32.7) | 1055 (32.7) | 861 (33.6) |
| Rarely (1/mo) | 659 (18.6) | 646 (18.8) | 511 (18.8) |
| Never | 1124 (24.7) | 1074 (24.4) | 802 (23.3) |
| **Feel sleepy** |  |  |  |
| Almost always (16-30/mo) | 164 (4.0) | 158 (4.0) | 121 (3.8) |
| Often (5-15/mo) | 386 (11.0) | 374 (11.0) | 304 (10.9) |
| Sometimes (2-4/mo) | 953 (27.4) | 928 (27.6) | 747 (28.1) |
| Rarely (1/mo) | 875 (25.8) | 852 (25.8) | 690 (26.5) |
| Never | 1346 (31.7) | 1294 (31.6) | 985 (30.7) |
| **Take sleeping pills** |  |  |  |
| Almost always (16-30/mo) | 120 (3.9) | 117 (4.0) | 83 (3.8) |
| Often (5-15/mo) | 78 (2.5) | 74 (2.4) | 57 (2.4) |
| Sometimes (2-4/mo) | 169 (5.1) | 168 (5.2) | 126 (5.0) |
| Rarely (1/mo) | 144 (4.3) | 142 (4.3) | 116 (4.3) |
| Never | 3216 (84.2) | 3108 (84.0) | 2467 (84.5) |
| **Sleep breathing problems** |  |  |  |
| **Snoring** |  |  |  |
| Frequently (5+/wk) | 977 (32.0) | 945 (31.8) | 740 (31.6) |
| Occasionally (3-4/wk) | 548 (17.4) | 540 (17.5) | 436 (17.5) |
| Rarely (1-2/wk) | 633 (20.3) | 954 (20.8) | 515 (21.4) |
| Never | 1097 (30.4) | 1801 (29.9) | 820 (29.4) |
| **Snort** |  |  |  |
| Frequently (5+/wk) | 158 (5.4) | 152 (5.4) | 119 (5.2) |
| Occasionally (3-4/wk) | 175 (5.2) | 173 (5.3) | 132 (5.3) |
| Rarely (1-2/wk) | 273 (8.2) | 269 (8.4) | 213 (8.1) |
| Never | 2837 (81.1) | 2745 (80.9) | 2179 (81.4) |
| **Sleep leg problems** |  |  |  |
| **Leg cramps** |  |  |  |
| Almost always (16-30/mo) | 37 (1.1) | 37 (1.1) | 26 (1.1) |
| Often (5-15/mo) | 114 (3.1) | 111 (3.2) | 72 (2.6) |
| Sometimes (2-4/mo) | 396 (10.3) | 386 (10.4) | 296 (10.0) |
| Rarely (1/mo) | 419 (11.3) | 408 (11.4) | 306 (10.9) |
| Never | 2759 (74.1) | 2665 (73.9) | 2148 (75.4) |
| **Leg jerks** |  |  |  |
| Almost always (16-30/mo) | 51 (1.8) | 50 (1.9) | 41 (1.9) |
| Often (5-15/mo) | 87 (2.5) | 86 (2.6) | 68 (2.6) |
| Sometimes (2-4/mo) | 283 (8.1) | 273 (8.0) | 220 (8.1) |
| Rarely (1/mo) | 268 (8.5) | 255 (8.3) | 205 (8.6) |
| Never | 3019 (79.1) | 2927 (79.2) | 2301 (78.8) |
| **Sleep Disorders** |  |  |  |
| **Any sleep disorder** |  |  |  |
| Yes | 166 (5.0) | 159 (5.0) | 115 (4.8) |
| No | 3558 (95.0) | 3447 (95.0) | 2732 (95.2) |
| **Apnea** |  |  |  |
| Yes | 90 (3.0) | 89 (3.1) | 63 (2.9) |
| No | 3627 (97.0) | 3513 (96.9) | 2780 (97.1) |
| **Insomnia** |  |  |  |
| Yes | 34 (0.7) | 33 (0.7) | 23 (0.6) |
| No | 3693 (99.3) | 3576 (99.3) | 2826 (99.4) |
| **Restless leg syndrome** |  |  |  |
| Yes | 8 (0.2) | 7 (0.2) | 2 (0.1) |
| No | 3719 (99.8) | 3602 (99.8) | 2847 (99.9) |
| **Other disorder** |  |  |  |
| Yes | 26 (0.9) | 25 (0.9) | 22 (1.0) |
| No | 3701 (99.1) | 3584 (99.1) | 2827 (99.0) |

^a^ Percentages are weighted

**Table S3** Spearman correlation coefficient ^a^ among sleep variables, NHANES 2005-2006

|  | **Sleep duration** | **Sleep quality index** | **How long to fall asleep** | **Have trouble falling asleep** | **Wake up at night** | **Wake up too early** | **Unrest during the day** | **Overly sleepy** | **Not enough sleep** | **Take sleeping pills** | **Snoring** | **Snort** | **Leg jerks** | **Leg cramps** |
| --- | --- | --- | --- | --- | --- | --- | --- | --- | --- | --- | --- | --- | --- | --- |
| **Sleep duration** | 1.00 | -0.28 | 0.12 | 0.19 | 0.19 | 0.19 | 0.19 | 0.19 | 0.35 | 0.02 | 0.12 | 0.09 | 0.08 | 0.07 |
| **Sleep quality index** |  | 1.00 | -0.42 | -0.50 | -0.45 | -0.40 | -0.43 | -0.42 | -0.45 | -0.34 | -0.04 | -0.09 | -0.16 | -0.16 |
| **How long to fall asleep** |  |  | 1.00 | 0.50 | 0.29 | 0.21 | 0.16 | 0.14 | 0.14 | 0.17 | -0.05 | -0.01 | 0.07 | 0.05 |
| **Have trouble falling asleep** |  |  |  | 1.00 | 0.55 | 0.41 | 0.36 | 0.32 | 0.36 | 0.29 | -0.01 | 0.05 | 0.20 | 0.14 |
| **Wake up at night** |  |  |  |  | 1.00 | 0.66 | 0.38 | 0.33 | 0.36 | 0.23 | 0.05 | 0.05 | 0.20 | 0.19 |
| **Wake up too early** |  |  |  |  |  | 1.00 | 0.32 | 0.28 | 0.34 | 0.17 | 0.05 | 0.05 | 0.17 | 0.17 |
| **Unrest during the day** |  |  |  |  |  |  | 1.00 | 0.70 | 0.61 | 0.17 | 0.05 | 0.10 | 0.19 | 0.17 |
| **Overly sleepy** |  |  |  |  |  |  |  | 1.00 | 0.57 | 0.17 | 0.06 | 0.08 | 0.19 | 0.16 |
| **Not enough sleep** |  |  |  |  |  |  |  |  | 1.00 | 0.17 | 0.05 | 0.10 | 0.17 | 0.12 |
| **Take sleeping pills** |  |  |  |  |  |  |  |  |  | 1.00 | 0.00 | 0.04 | 0.14 | 0.08 |
| **Snoring** |  |  |  |  |  |  |  |  |  |  | 1.00 | 0.37 | 0.10 | 0.08 |
| **Snort** |  |  |  |  |  |  |  |  |  |  |  | 1.00 | 0.13 | 0.14 |
| **Leg jerks** |  |  |  |  |  |  |  |  |  |  |  |  | 1.00 | 0.37 |
| **Leg cramps** |  |  |  |  |  |  |  |  |  |  |  |  |  | 1.00 |

^a^ weighted

**Table S4** Spearman correlation coefficient ^a^ among measurements of adiposity, by sex, NHANES 2005-2006

|  | **BMI** | **Waist circumference** | **% body fat** | **Android/gynoid fat ratio** |
| --- | --- | --- | --- | --- |
| **BMI** | 1 | 0.90 | 0.59 | 0.50 |
| **Waist circumference** |  | 1 | 0.46 | 0.66 |
| **% body fat** |  |  | 1 | 0.00 |
| **Android/gynoid fat ratio** |  |  |  | 1 |

^a^ weighted

**Table S5** Study participant characteristics by snoring, NHANES 2005-2006

|  | **Snoring** | | | |
| --- | --- | --- | --- | --- |
|  | **Frequently (5+/wk)** | **Occasionally (3-4/wk)** | **Rarely**  **(1-2/wk)** | **Never** |
| Age, year, mean (SD) | 44.7 (14.5) | 43.2 (14.4) | 42.4 (15.7) | 39.2 (17.1) |
| Female, % | 33.3 | 45.3 | 53.3 | 59.3 |
| Non-Hispanic White, % | 72.1 | 70.7 | 74.4 | 66.1 |
| Non-Hispanic Black, % | 10.0 | 13.5 | 10.8 | 13.1 |
| Less than high school grad, % | 15.7 | 14.5 | 12.3 | 16.1 |
| Current smoker, % | 27.0 | 22.0 | 19.1 | 18.7 |
| Former smoker, % | 25.3 | 19.4 | 25.5 | 16.8 |
| Alcohol consumption, > 1 drink/day, % | 19.5 | 14.1 | 11.5 | 9.7 |
| Fat intake, mean (SD), gram/kcal | 37.5 (8.7) | 37.9 (8.3) | 38.0 (8.2) | 39.2 (8.5) |
| Carbohydrate intake, mean (SD), gram/kcal | 119 (25) | 121 (23) | 121 (23) | 124 (23) |
| Total caloric intake, kcal | 2335 (924) | 2227 (796) | 2168 (816) | 2117 (862) |
| History of diabetes, % | 6.5 | 7.5 | 4.7 | 3.9 |
| Sit during the day and not walk about much, % | 23.8 | 17.7 | 20.5 | 19.5 |

Percentages, means and standard deviations are weighted

**Table S6** Study participant characteristics by sleep quality, NHANES 2005-2006

|  | **Sleep quality score** | | |
| --- | --- | --- | --- |
|  | **2+** | **1** | **0** |
| Age, year, mean (SD) | 42.3 (15.6) | 42.7 (16.7) | 42.6 (15.9) |
| Female, % | 61.2 | 52.6 | 48.4 |
| Non-Hispanic White, % | 70.5 | 63.6 | 70.5 |
| Non-Hispanic Black, % | 12.6 | 16.9 | 11.1 |
| Less than high school grad, % | 20.6 | 16.8 | 14.6 |
| Current smoker, % | 30.9 | 27.4 | 19.9 |
| Former smoker, % | 19.3 | 23.2 | 21.7 |
| Alcohol consumption, > 1 drink/day, % | 15.0 | 12.7 | 13.7 |
| Fat intake, mean (SD), gram/kcal | 36.7 (8.8) | 38.1 (8.3) | 37.3 (8.4) |
| Carbohydrate intake, mean (SD), gram/kcal | 123 (23) | 121 (24) | 121 (24) |
| Total caloric intake, kcal | 2146 (959) | 2182 (826) | 2212 (864) |
| History of diabetes, % | 7.8 | 8.3 | 4.8 |
| Sit during the day and not walk about much, % | 26.7 | 20.6 | 19.9 |

Percentages, means and standard deviations are weighted

**Table S7** Multivariate ^a^ associations between sleep characteristics and measurements of adiposity in men, NHANES 2005-2006

|  | **BMI (kg/m2)** | **% body fat** | **Waist circumference (cm)** | **Android/Gynoid fat ratio** |
| --- | --- | --- | --- | --- |
|  |  |  |  |  |
| **Sleep Duration** |  |  |  |  |
| ≤5 hr | 2.14 (0.93, 3.35) | 1.48 (0.51, 2.45) | 4.58 (1.71, 7.45) | 0.02 (-0.02, 0.06) |
| 6 hr | 1.10 (0.40, 1.80) | 0.54 (-0.15, 1.24) | 1.99 (0.47, 3.52) | 0.00 (-0.03, 0.03) |
| 7 hr | 0.45 (-0.26, 1.16) | 0.47 (-0.07, 1.00) | 0.96 (-0.46, 2.39) | -0.01 (-0.03, 0.02) |
| 8 hr | ref | ref | ref | ref |
| ≥9 hr | 0.24 (-1.16, 1.63) | 1.06 (-0.39, 2.52) | -0.35 (-2.72, 2.02) | -0.02 (-0.06, 0.01) |
| *p for trend* | *0.001* | *0.07* | *0.004* | *0.08* |
| **Sleep Quality** |  |  |  |  |
| **Sleep quality index** |  |  |  |  |
| 2+ | 1.19 (0.34, 2.04) | 1.18 (0.44, 1.93) | 2.82 (0.87, 4.77) | 0.04 (0.00, 0.08) |
| 1 | 0.93 (0.30, 1.57) | 0.87 (0.22, 1.53) | 1.59 (0.06, 3.12) | 0.02 (-0.01, 0.04) |
| 0 | ref | ref | ref | ref |
| *p for trend* | *0.002* | *0.0004* | *0.001* | *0.01* |
| **How long to fall asleep** |  |  |  |  |
| >= 1hr | 0.61 (-0.40, 1.63) | 0.75 (-0.30, 1.80) | 1.64 (-0.75, 4.04) | 0.03 (0.00, 0.07) |
| 30-<1 hr | -0.25 (-1.09, 0.59) | 0.25 (-0.56, 1.07) | -0.52 (-2.38, 1.33) | 0.01 (-0.01, 0.03) |
| 15-<30 min | -0.21 (-1.00, 0.58) | 0.10 (-0.58, 0.79) | -0.54 (-2.64, 1.55) | -0.01 (-0.03, 0.02) |
| <15 min | ref | ref | ref | ref |
| *p for trend* | *0.61* | *0.20* | *0.50* | *0.11* |
| **Have trouble falling asleep** |  |  |  |  |
| Almost always (16-30/mo) | 0.71 (-0.78, 2.21) | 0.56 (-1.05, 2.17) | 1.63 (-1.84, 5.11) | 0.04 (0.01, 0.08) |
| Often (5-15/mo) | -0.21 (-1.35, 0.93) | 0.12 (-0.92, 1.16) | -0.56 (-3.07, 1.95) | -0.01 (-0.04, 0.02) |
| Sometimes (2-4/mo) | 0.18 (-0.43, 0.78) | 0.13 (-0.75, 1.01) | 0.41 (-1.72, 2.54) | 0.01 (-0.03, 0.04) |
| Rarely (1/mo) | -0.15 (-0.68, 0.38) | -0.17 (-1.01, 0.68) | -0.19 (-1.91, 1.52) | 0.00 (-0.03, 0.03) |
| Never | ref | ref | ref | ref |
| *p for trend* | *0.54* | *0.56* | *0.60* | *0.25* |
| **Wake up at night** |  |  |  |  |
| Almost always (16-30/mo) | 0.39 (-0.43, 1.21) | 0.73 (-0.22, 1.68) | 0.88 (-1.02, 2.78) | 0.02 (-0.03, 0.06) |
| Often (5-15/mo) | -0.05 (-1.12, 1.01) | 0.14 (-1.10, 1.38) | -0.24 (-2.7, 2.21) | 0.00 (-0.02, 0.02) |
| Sometimes (2-4/mo) | -0.36 (-1.00, 0.28) | -0.51 (-1.02, 0.00) | -1.15 (-2.69, 0.40) | -0.02 (-0.05, 0.01) |
| Rarely (1/mo) | -0.46 (-1.17, 0.25) | -0.16 (-0.86, 0.54) | -1.21 (-3.03, 0.62) | -0.02 (-0.05, 0.00) |
| Never | ref | ref | ref | ref |
| *p for trend* | *0.94* | *0.74* | *0.88* | *0.91* |
| **Wake up too early** |  |  |  |  |
| Almost always (16-30/mo) | 0.49 (-0.92, 1.89) | 0.98 (-0.66, 2.63) | 0.82 (-2.58, 4.22) | -0.02 (-0.08, 0.04) |
| Often (5-15/mo) | 0.06 (-0.61, 0.74) | -0.03 (-0.74, 0.69) | 0.29 (-1.62, 2.20) | 0.01 (-0.03, 0.04) |
| Sometimes (2-4/mo) | -0.18 (-0.80, 0.43) | 0.07 (-0.37, 0.51) | -0.52 (-1.69, 0.64) | 0.00 (-0.03, 0.03) |
| Rarely (1/mo) | 0.23 (-0.44, 0.90) | 0.38 (-0.24, 1.00) | 0.70 (-1.02, 2.41) | -0.01 (-0.03, 0.02) |
| Never | ref | ref | ref | ref |
| *p for trend* | *0.67* | *0.33* | *0.76* | *0.85* |
| **Unrest during the day** |  |  |  |  |
| Almost always (16-30/mo) | 1.53 (0.42, 2.63) | 1.73 (0.56, 2.91) | 3.15 (0.78, 5.51) | 0.02 (-0.02, 0.07) |
| Often (5-15/mo) | 0.41 (-0.50, 1.32) | 0.02 (-0.77, 0.81) | 0.96 (-1.21, 3.13) | -0.01 (-0.03, 0.02) |
| Sometimes (2-4/mo) | -0.12 (-0.73, 0.48) | -0.22 (-0.86, 0.42) | -0.34 (-1.89, 1.21) | -0.01 (-0.03, 0.01) |
| Rarely (1/mo) | -0.01 (-0.72, 0.71) | 0.03 (-0.71, 0.78) | -0.03 (-1.55, 1.50) | -0.02 (-0.05, 0.00) |
| Never | ref | ref | ref | ref |
| *p for trend* | *0.04* | *0.10* | *0.07* | *0.38* |
| **Not enough sleep** |  |  |  |  |
| Almost always (16-30/mo) | 1.40 (0.26, 2.53) | 0.63 (-0.45, 1.71) | 2.98 (1.09, 4.86) | 0.03 (-0.01, 0.08) |
| Often (5-15/mo) | 0.35 (-0.08, 0.78) | -0.17 (-0.88, 0.54) | 0.43 (-0.86, 1.73) | -0.01 (-0.03, 0.02) |
| Sometimes (2-4/mo) | -0.05 (-0.79, 0.68) | -0.41 (-1.08, 0.26) | 0.12 (-1.54, 1.78) | -0.02 (-0.04, 0.00) |
| Rarely (1/mo) | 0.17 (-0.62, 0.96) | 0.27 (-0.56, 1.11) | 0.88 (-0.69, 2.46) | 0.00 (-0.04, 0.03) |
| Never | ref | ref | ref | ref |
| *p for trend* | *0.04* | *0.92* | *0.09* | *0.49* |
| **Overly sleepy** |  |  |  |  |
| Almost always (16-30/mo) | 1.68 (0.46, 2.91) | 1.91 (0.61, 3.21) | 3.14 (-0.07, 6.35) | 0.03 (-0.02, 0.08) |
| Often (5-15/mo) | 0.61 (-0.33, 1.55) | -0.04 (-0.96, 0.88) | 1.48 (-0.40, 3.36) | 0.01 (-0.02, 0.03) |
| Sometimes (2-4/mo) | 0.23 (-0.28, 0.75) | 0.03 (-0.57, 0.64) | 0.23 (-1.08, 1.55) | -0.02 (-0.04, 0.00) |
| Rarely (1/mo) | 0.10 (-0.70, 0.89) | 0.09 (-0.72, 0.89) | 0.39 (-1.41, 2.18) | -0.01 (-0.03, 0.02) |
| Never | ref | ref | ref | ref |
| *p for trend* | *0.03* | *0.21* | *0.07* | *0.81* |
| **Take sleeping pills** |  |  |  |  |
| Almost always (16-30/mo) | -0.05 (-1.37, 1.27) | -0.25 (-1.82, 1.33) | 0.50 (-2.49, 3.49) | 0.03 (-0.03, 0.09) |
| Often (5-15/mo) | 1.27 (-1.29, 3.83) | 1.73 (-0.57, 4.03) | 2.81 (-2.99, 8.62) | 0.01 (-0.05, 0.07) |
| Sometimes (2-4/mo) | 0.16 (-0.94, 1.25) | -0.11 (-1.06, 0.83) | -0.4 (-2.49, 1.69) | -0.01 (-0.03, 0.02) |
| Rarely (1/mo) | -0.30 (-1.71, 1.11) | -0.03 (-1.45, 1.39) | 0.24 (-3.49, 3.98) | -0.02 (-0.06, 0.03) |
| Never | ref | ref | ref | ref |
| *p for trend* | *0.67* | *0.74* | *0.58* | *0.47* |
| **Sleep breathing problems** |  |  |  |  |
| **Snoring** |  |  |  |  |
| Frequently (5+/wk) | 4.56 (3.56, 5.55) | 3.57 (2.69, 4.45) | 10.29 (8.09, 12.48) | 0.10 (0.07, 0.13) |
| Occasionally (3-4/wk) | 2.80 (2.01, 3.60) | 2.46 (1.83, 3.10) | 7.18 (5.14, 9.23) | 0.09 (0.07, 0.12) |
| Rarely (1-2/wk) | 1.85 (0.88, 2.83) | 1.61 (0.84, 2.38) | 4.52 (2.29, 6.75) | 0.03 (0.01, 0.06) |
| Never | ref | ref | ref | ref |
| *p for trend* | *<.0001* | *<.0001* | *<.0001* | *<.0001* |
| **Snort** |  |  |  |  |
| Frequently (5+/wk) | 3.63 (2.60, 4.66) | 2.68 (1.33, 4.03) | 8.20 (6.56, 9.83) | 0.07 (0.01, 0.12) |
| Occasionally (3-4/wk) | 2.42 (1.23, 3.61) | 2.15 (1.01, 3.30) | 5.21 (2.71, 7.71) | 0.06 (0.01, 0.11) |
| Rarely (1-2/wk) | 1.30 (0.42, 2.19) | 1.25 (0.32, 2.18) | 3.23 (1.39, 5.07) | 0.01 (-0.04, 0.00) |
| Never | ref | ref | ref | ref |
| *p for trend* | *<.0001* | *0.0001* | *<.0001* | *0.004* |
| **Sleep leg problems** |  |  |  |  |
| **Leg cramps** |  |  |  |  |
| Almost always (16-30/mo) | 0.02 (-2.11, 2.16) | 0.91 (-1.45, 3.27) | 1.30 (-3.86, 6.46) | 0.07 (-0.01, 0.15) |
| Often (5-15/mo) | 1.49 (-0.56, 3.54) | 1.87 (-0.35, 4.08) | 3.14 (-2.15, 8.43) | 0.01 (-0.05, 0.07) |
| Sometimes (2-4/mo) | 0.26 (-0.50, 1.02) | 0.89 (0.02, 1.76) | 1.38 (-0.40, 3.16) | 0.01 (-0.03, 0.05) |
| Rarely (1/mo) | 0.58 (0.12, 1.05) | 0.92 (0.42, 1.41) | 2.44 (1.05, 3.82) | 0.01 (-0.02, 0.04) |
| Never | ref | ref | ref | ref |
| *p for trend* | *0.09* | *0.01* | *0.03* | *0.22* |
| **Leg jerks** |  |  |  |  |
| Almost always (16-30/mo) | 0.49 (-2.86, 3.84) | 0.14 (-2.94, 3.21) | 1.25 (-4.78, 7.29) | 0.07 (0.00, 0.15) |
| Often (5-15/mo) | -0.42 (-1.52, 0.68) | -0.83 (-2.33, 0.67) | -0.73 (-3.32, 1.86) | -0.03 (-0.09, 0.03) |
| Sometimes (2-4/mo) | -0.85 (-1.71, 0.01) | -0.65 (-1.61, 0.32) | -1.36 (-3.40, 0.67) | 0.00 (-0.03, 0.04) |
| Rarely (1/mo) | -0.03 (-0.95, 0.89) | -0.19 (-0.99, 0.61) | -0.25 (-2.55, 2.05) | -0.03 (-0.06, 0.00) |
| Never | ref | ref | ref | ref |
| *p for trend* | *0.50* | *0.34* | *0.61* | *0.65* |
| **Sleep Disorders** |  |  |  |  |
| **Any sleep disorder** |  |  |  |  |
| Yes | 2.69 (1.74, 3.64) | 1.68 (0.52, 2.83) | 7.52 (4.94, 10.11) | 0.08 (0.05, 0.12) |
| No | ref | ref | ref | ref |
| **Apnea** |  |  |  |  |
| Yes | 4.29 (3.13, 5.44) | 3.14 (1.45, 4.83) | 11.31 (8.69, 13.93) | 0.11 (0.05, 0.17) |
| No | ref | ref | ref | ref |
| **Insomnia** |  |  |  |  |
| Yes | -1.41 (-4.87, 2.06) | -1.34 (-4.82, 2.15) | -1.50 (-9.63, 6.61) | 0.03 (-0.05, 0.10) |
| No | ref | ref | ref | ref |
| **Restless leg syndrome** |  |  |  |  |
| Yes | 6.34 (0.53, 12.14) | 4.48 (-8.35, 17.31) | 13.17 (-4.24, 30.58) | 0.13 (0.09, 0.16) |
| No | ref | ref | ref | ref |
| **Other disorder** |  |  |  |  |
| Yes | -0.20 (-3.26, 2.85) | -0.78 (-4.81, 3.24) | 1.27 (-7.80, 10.34) | 0.04 (-0.08, 0.15) |
| No | ref | ref | ref | ref |

^b^ adjusted for age, race/ethnicity, smoking status, alcohol drinking, education, intakes of fat, carbohydrate and total calories, physical activity and diabetes
